# Miocene Diversification in the Savannahs Precedes Tetraploid Rainforest Radiation in the African Tree Genus *Afzelia* (Detarioideae, Fabaceae)

**DOI:** 10.3389/fpls.2020.00798

**Published:** 2020-06-17

**Authors:** Armel S. L. Donkpegan, Jean-Louis Doucet, Olivier J. Hardy, Myriam Heuertz, Rosalía Piñeiro

**Affiliations:** ^1^Forest is Life, TERRA Teaching and Research Centre, Gembloux Agro-Bio Tech, University of Liège, Gembloux, Belgium; ^2^Evolutionary Biology and Ecology Unit, Faculté des Sciences, Université Libre de Bruxelles, Brussels, Belgium; ^3^INRAE, BFP, University of Bordeaux, Villenave d’Ornon, France; ^4^INRAE, BIOGECO, University of Bordeaux, Cestas, France; ^5^Department of Geography, College of Life and Environmental Sciences, University of Exeter, Exeter, United Kingdom; ^6^Evolutionary Genomics, Centre for Geogenetics – Natural History Museum of Denmark, Copenhagen, Denmark

**Keywords:** *Afzelia*, Leguminosae (Detarioideae), high-throughput sequencing, phylogenomics, coalescent approaches, biome shift, molecular dating, species trees

## Abstract

The dating of diversification events, including transitions between biomes, is key to elucidate the processes that underlie the assembly and evolution of tropical biodiversity. *Afzelia* is a widespread genus of tropical trees, threatened by exploitation for its valuable timber, that presents an interesting system to investigate diversification events in Africa. Africa hosts diploid *Afzelia* species in the savannahs north and south of the Guineo-Congolian rainforest and autotetraploid species confined to the rainforest. Species delimitation and phylogenetic relationships among the diploid and tetraploid species remained unresolved in previous studies using small amounts of DNA sequence data. We used genotyping-by-sequencing in the five widespread *Afzelia* species in Africa, the savannah species *A. africana* and *A. quanzensis* and the rainforest species *A. bipindensis*, *A. pachyloba*, and *A. bella*. Maximum likelihood and coalescent approaches resolved all species as monophyletic and placed the savannah and rainforest taxa into two separate clades corresponding to contrasted ploidy levels. Our data are thus compatible with a single biome shift in *Afzelia* in Africa, although we were unable to conclude on its direction. SNAPP calibrated species trees show that the savannah diploids started to diversify early, at 12 (9.09–14.89) Ma, which contrasts with a recent and rapid diversification of the rainforest tetraploid clade, starting at 4.22 (3.12 – 5.36) Ma. This finding of older diversification in a tropical savannah clade vs. its sister rainforest clade is exceptional; it stands in opposition to the predominant observation of young ages for savannahs lineages in tropical regions during the relatively recent expansion of the savannah biome.

## Introduction

The biogeographic history of the African flora has been marked by an overall trend toward continental aridification since the wet and warm conditions of the Paleocene (66 – 56 Ma, Senut et al., 2009; Jacobs et al., 2010). Paleobotanical evidence from the north of Africa suggests that rainforest was the most common biome during the Paleocene and the beginning of the Eocene (56 – 33.9 Ma, Jacobs, 2004; Jacobs et al., 2010). More open vegetation appeared in central Africa in the middle Eocene (47.8 – 38 Ma) concomitant with increased temperatures and aridification (Jacobs et al., 2010). A global cooling at the Eocene-Oligocene boundary (33 Ma) led to large-scale extinctions (Zachos et al., 2008; Jacobs et al., 2010) and the grass-dominated savannah biome began to expand in the middle Miocene (16 Ma, Jacobs, 2004), becoming a well established component of tropical vegetation from the late Miocene (ca. 8 Ma, Cerling et al., 1997). The alternation of cold/dry and hot/humid climates of the Miocene (23 – 5.3 Ma), Pliocene (5.3 – 2.6 Ma) and Pleistocene (2.6 – 0.01 Ma) has affected the distribution the major tropical biomes - rainforest, woodland and savannah – with repetitive phases of major expansion or contraction, resulting in the modern distribution of tropical African biomes (Sarnthein and Fenner, 1988; Morley, 2000; Plana, 2004; Salzmann and Hoelzmann, 2005; Anhuf et al., 2006; Miller and Gosling, 2014).

These historical contractions and expansions of the major African biomes have probably triggered biome shifts and diversification in the evolution of tropical plant lineages. Understanding and dating biome shifts is key to understanding the processes that underlie the assembly and evolution of African tropical biodiversity (Wiens and Donoghue, 2004), however, biome shifts have been little studied in the African floras. Multiple transitions from rainforests to dry forests/savannahs have been inferred in the diversification of the tree genus *Guibourtia* in Africa (Tosso et al., 2018). Similarly, three biome transitions from humid forest to dry or montane forests have been documented in the tree genus *Entandrophragma* (Meliaceae) along with ecological adaptations to drier habitat (Monthe et al., 2019). The literature suggests that most biome shifts in tropical Africa support the transition from closed habitats to open habitats (Holstein and Renner, 2011; Veranso-Libalah et al., 2018), which is congruent with paleobotanical evidence for rainforest to be ancient and savannahs to be a more recent biome (Jacobs et al., 2010).

In African forest trees, phylogenetics or population genetics studies have led to the discovery of many new species that could not *a priori* be distinguished based on morphological features (Koffi et al., 2010; Duminil et al., 2012; Heuertz et al., 2014; Daïnou et al., 2016; Ikabanga et al., 2017; Lissambou et al., 2018). High-throughput sequencing can facilitate the study of taxonomically difficult groups that contain closely related, weakly differentiated species. Sequencing large portions of the genome of non-model organisms can help generate resolved phylogenies of these complex groups. For non-model taxa, reduced representation sequencing methods such as genotyping-by-sequencing (GBS, – Elshire et al., 2011) can provide thousands of single nucleotide polymorphisms (SNPs) for phylogenetic analysis without prior knowledge of the genome (Eaton and Ree, 2013; Escudero et al., 2014; Hipp et al., 2014; Ariani et al., 2016; Nicotra et al., 2016; Fernández-Mazuecos et al., 2018).

**FIGURE 1:**
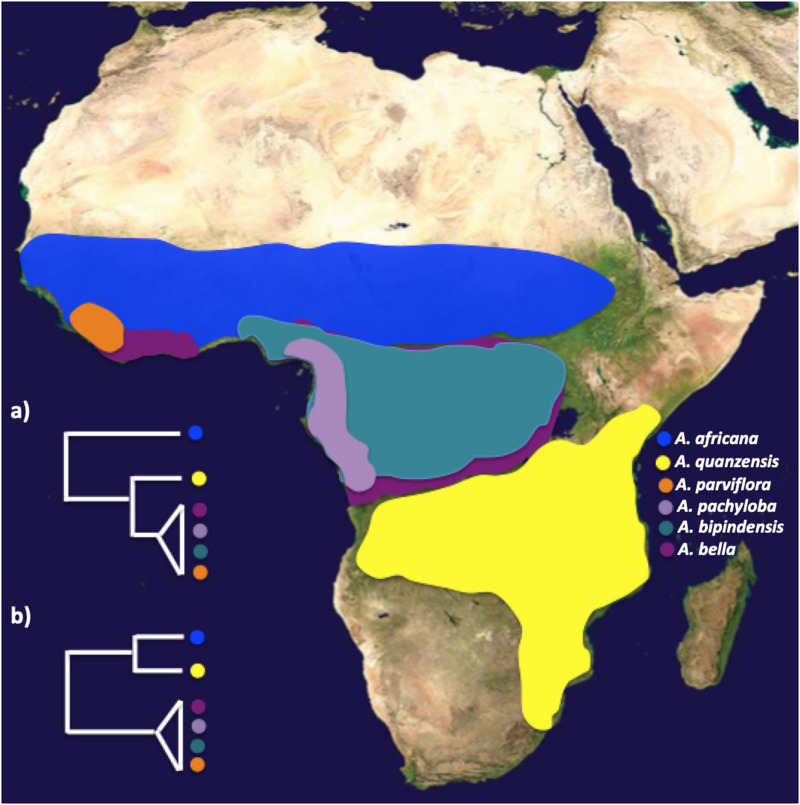
Biogeographic ranges of *Afzelia* sequenced in this study, and alternative phylogenetic relationships **(a,b)** recovered from a previous study (Donkpegan et al., 2017). Plastid data (*psbA*, *trnL*, *ndhF* and genome-wide SNPs via plastomes) suggest that savannah species are paraphyletic with respect to forest taxa **(a)**, whereas nuclear markers (ribosomal *ITS* and the single-copy *PEPC* E7 gene) recovered distinct savannah and tropical forest clades **(b)**. Map image: public domain from www.simplemappr.net.

*Afzelia* Smith (Detarioideae – Caesalpinioideae) is a widespread and taxonomically complex genus of valuable timber trees that provides an excellent opportunity to apply genomic tools for species delimitation and investigate the role played by biome shifts in species diversification in tropical Africa. *Afzelia* is a Paleotropical genus distributed in Sub-Saharan Africa, where it is known as “doussié,” and Southeast Asia (Donkpegan et al., 2014). The genus exhibits large morphological variability within and between species and can be considered a species complex (Donkpegan et al., 2014). At present, most taxonomists agree that it contains 11 species (Chevalier, 1940; Léonard, 1950; Institut National pour l’Etude Agronomique du Congo-belge [INEAC], 1952; Aubréville, 1959, 1968, 1970; Satabié, 1994). Seven species occur in sub-Saharan Africa: five of them are widely distributed, the savannah species *A. africana* Sm. ex Pers., and *A. quanzensis* Welw., and the rainforest species *A. bipindensis* Harms, *A. bella* Harms, *A. pachyloba* Harms (Figure 1); and two are local endemics, *A. parviflora* (Vahl) Hepper occurring in rainforest habitat in West Africa, and *A. peturei* De Wild, probably the least documented species of the *Afzelia* clade in Africa, being found in the transition zone between the rainforest and the Zambesian savannah. The remaining four species, *A. xylocarpa* (Kurz) Craib, *A. rhomboidea* (Blanco) S. Vidal, *A. javanica* (Miq) J. Léonard and *A. palembanica* Baker, occur in Southeast Asia in scattered locations in dry, mixed deciduous or evergreen dipterocarp forest. Based on a fossil attributed to *Afzelia* discovered in the Guang River flora in north-western Ethiopia and dating from the Late Oligocene (27.23 Ma, Pan et al., 2010), it is likely that the genus originated in Africa and that it dispersed subsequently into tropical Asia. Most *Afzelia* species are categorized as vulnerable according to the International Union for the Conservation of Nature (IUCN) Red List because they are substantially exploited for the international timber market (International Union for Conservation of Nature and Natural Resources [IUCN], 2012).

In the evolution of *Afzelia* species in Africa, biome shifts seem to have taken place in association with ploidy levels. The rainforest species *A. bipindensis*, *A. bella*, *A. pachyloba*, and *A. parviflora* – sympatrically distributed across the Guineo-Congolian rainforest – have recently been shown to be autotetraploids using nuclear microsatellites and flow cytometry, whereas the savannah species *A. africana* and *A. quanzensis* – situated north and south of the Guineo-Congolian rainforest, respectively – are diploids (Donkpegan et al., 2015). However, based on Sanger sequencing of two nuclear (nDNA) and three plastid (pDNA) regions, and on full plastome sequences for each of the species, the phylogenetic relationships in the polyploid complex remain uncertain. First, the phylogenetic relationships between the forest and the savannah species were not resolved. Therefore, it remains uncertain whether the forest-savannah transitions have happened once or multiple times in the evolution of the genus. There was cyto-nuclear incongruence with respect to the placement of *A. quanzensis*: pDNA placed the savannah species *A. quanzensis* as sister to the tetraploid forest clade, whereas nuclear markers identified the savannah and the forest species as two monophyletic sister clades (Figures 1a,b; Donkpegan et al., 2017). Second, the forest taxa showed little genetic differentiation and displayed extensive plastid and nDNA haplotype sharing across species based on few genetic markers, which could be due to incorrect taxonomy, recent speciation with large effective population sizes or ongoing hybridization (Pennington and Lavin, 2016), whereas the savannah species were genetically well differentiated (Donkpegan et al., 2017, 2020). A phylogeny based on genome-wide genetic markers has the potential to improve the taxonomic classification and our understanding of the evolutionary history of this genus of economically important African trees, including the history of biome shifts between forests and savannahs, and shed light on the speciation process in the rainforest taxa.

In this study, we used GBS to sequence the five most abundant species of the genus *Afzelia* in Africa (*A. africana*, *A. quanzensis*, *A. bipindensis*, *A. bella*, and *A. pachyloba*) in order to assess the phylogenetic relationships among them using multiple methods and datasets. We addressed the following questions.

1. Given the previously unresolved phylogeny, can genome-wide genetic markers provide additional insights into the phylogenetic relationships between diploid savannah and tetraploid rainforest species in *Afzelia*?
2. If so, can molecular dating of the phylogeny inform on which biome shifts occurred during the diversification of *Afzelia* in Africa?
3. Given the extensive haplotype sharing previously observed, can multiple genomic markers delimit species and provide insights into the timing of diversification and/or hybridization in the polyploid complex of rainforest *Afzelia* taxa?

We find strong support for the delimitation of the investigated *Afzelia* species and for phylogenetic relationships between species. This study represents the most comprehensive phylogenomic evaluation of *Afzelia* to date.

## Materials and Methods

### Sampling, DNA Extraction, Genomic Libraries and Sequencing

We used 41 accessions of *Afzelia* and six accessions (Supplementary Material S1) of other Leguminosae species as outgroups. Our sampling represents the five widely distributed species of the genus *Afzelia* in Africa (Donkpegan, 2017): the diploid savannah species *A. africana* (12 accessions) and *A. quanzensis* (7 accessions) and the tetraploid rainforest species *A. bipindensis* (14 accessions), *A. bella* (4 accessions) *A. pachyloba* Harms (4 accessions). Two species of *Afzelia* (*A. parviflora* and *A. peturei*), which have very restricted ranges, were not included in this study due to lack of recently collected plant material required for GBS. The outgroups were chosen according to the latest available phylogenies in the legume family (Bruneau et al., 2008; LPWG, 2017): namely *Scorodophloeus zenkeri* Harms (one accession), *Prioria balsamifera* (Vermoesen) Breteler (2 accessions), *Prioria oxyphylla* (Harms) Breteler (2 accessions), *Peltogyne sp*. (one accession) and the putative sister species to the *Afzelia* clade, *Intsia bijuga* (Colebr.) Kuntze (one accession). Metadata on all accessions are given in Supplementary Material S1.

DNA was extracted from silica-dried leaves collected in the field and four recent herbarium specimens (National Herbarium of the Netherlands Wageningen, WAG; African Botanical Library of Université Libre de Bruxelles, BRLU; and the Botanic Garden Meise, BR). For each accession, total genomic DNA was extracted using a CTAB protocol (Doyle and Doyle, 1987) and further purified using the QIAquick method (Qiagen, Venlo, Netherlands). We then quantified and controlled the quality of DNA using a QIAxcel (Qiagen). Before library construction, DNA extracts were further purified using a ZR-96 DNA Clean up kit (Zymo Research, Orange, CA, United States) to remove secondary metabolites. DNA quality was checked on a 1.5% agarose gel and DNA quantity was measured with Qbit HS (Thermo Fisher Scientific, Karlsruhe, Germany). To reach the high concentrations required for the genotyping-by-sequencing (GBS) protocol, two extractions per individual sample were pooled at this second purification step whenever possible.

Overall, 180 GBS libraries were built and sequenced on two Illumina lanes (HiSeq2000, San Diego, CA, United States), using 100-bp Single Read chemistry. Given the large genome sizes of our study species (4.9 – 5.0 pg in the diploids and 8.5 – 9.9 in the tetraploids, Donkpegan et al., 2015), two or three independent GBS libraries per individual were built and sequenced for the diploid and tetraploid individuals, respectively. GBS was performed at the Institute for Genomic Diversity and Computational Biology Service Unit at Cornell University (Ithaca, NY, United States) according to a published protocol (Elshire et al., 2011). To select the best enzyme for the GBS protocol, one microgram of DNA of *Afzelia bipindensis* was used to build test libraries using three different enzymes: *Ape*KI (4.5-base cutter), *Eco*T22I and *Pst*I (both 6-base cutters). Libraries were checked for appropriate fragment sizes (<500 bp) and distribution on an Experion automated electrophoresis system (Bio-Rad Laboratories, Hercules, CA, United States). The enzyme *Eco*T22I gave appropriate fragment sizes (<500 bp) and was selected.

### Bioinformatics Analyses

#### *De novo* Assembly of Reference Sequence

Single-end reads were checked for quality using FastQC 0.11.5 software (Andrews, 2010). Fastq-formatted GBS data was demultiplexed with Saber software^1^. Low quality bases and adapter contamination were removed with TRIMMOMATIC version 0.33 (Bolger et al., 2014) with the following options: ILLUMINACLIP 2:30:10, LEADING 3, TRAILING 3, SLIDINGWINDOW 4:15, MINLEN 36. The trimmed reads of all *Afzelia* accessions were *de novo* assembled using PyRAD v.3.0.2 software (Eaton and Ree, 2013, see parameters file in Supplementary Material S2): sequences were clustered within individuals using VSEARCH allowing for indels and nucleotide polymorphisms and assuming a minimum similarity rate of 85% (Rognes et al., 2016). Consensus allele sequences of each cluster (GBS locus) were generated for each individual based on the jointly estimated heterozygosity (H) and the error rate (E). A two-step approach was used to enrich the final dataset in *Afzelia* loci, and genotype all accessions including outgroups for the same loci. In a first step, clustering was performed using all *Afzelia* accessions in order to build a reference catalog with a minimum similarity rate of 85%. Preliminary genotyping was conducted with PyRAD assuming a minimum depth of 8 and a maximum of four shared polymorphic sites across individuals, to minimize the inclusion of paralogs. In a second step, the best *Afzelia* accession was selected, based on the lowest amount of missing data, as the reference catalog of GBS loci in PyRAD for read mapping and final SNP calling (next section) of all accessions, including outgroups.

#### SNP Discovery and Genotyping

The trimmed reads of all accessions, including outgroups, were then aligned to the reference sequence using the Burrows-Wheeler Aligner BWA mem 0.7.5a-r405 (Li and Durbin, 2009) with -M and -B 4 options, to generate SAM files. SAM files were processed using SAMtools 0.1.17 (Li et al., 2009) and Picard Tools v1.96^2^ to convert from SAM to BAM (Binary Alignment Map) (Sam Format Converter module), sorting the BAM files by position (Sort Sam module) and adding read groups (AddOrReplaceReadGroups module). The resulting BAM files were used as input for Genome Analysis Toolkit (GATK) v3.7 (Depristo et al., 2011). HaplotypeCaller variant discovery was run using emitRefConfidence GVCF mode separately for each sample (Depristo et al., 2011) and GenotypeGVCFs was run on the combined GVCF files to call and genotype SNPs. Although both diploids and tetraploids were present in the dataset, we used a diploid genotyping model in GATK to facilitate analyses involving both ploidy levels and because this practice is known to provide robust results, with only minor loss in numbers of SNPs in the tetraploids (Anderson et al., 2017). VCFtoolsv0.1.15 (Danecek et al., 2011) was used to filter out indels and non-biallelic variants. Different filter criteria were tested for missing data per sample and per SNP (see section “Results”). To evaluate the utility of GBS to infer phylogenetic relationships in *Afzelia*, we considered two separate datasets: the first containing all *Afzelia* samples with the outgroup taxa (hereafter *Afzelia with outgroups* dataset) and the second containing *Afzelia* samples only (hereafter *Afzelia dataset*). The filtered VCF files were converted to a fasta files containing a single consensus sequence per individual using PGDSpider version 2.1.1.5 (Lischer and Excoffier, 2012).

### Phylogenetic Analyses and Estimation of Divergence Times

For the *Afzelia with outgroups* dataset we applied Maximum Likelihood (ML) methods to perform phylogenetic analyses. ML analyses were conducted with the GTR + GAMMA substitution model and 100 bootstrap replicates running RAxML 7.2.6 (Stamatakis, 2014) with default parameters through the CIPRES Portal 2.1 (Miller, 2009)^3^. Phylogenetic trees were visualized in FigTree 1.4.3 (Rambaut, 2007). The analyses were repeated with ascertainment bias correction using the Lewis method to avoid overestimation of branch lengths and biases in the phylogeny when the number of non-variable sites is not known (Lewis, 2001). We then estimated divergence times within *Afzelia* based on the resulting phylogeny using Bayesian MCMC analysis implemented in BEAST 1.7.4 (Drummond et al., 2012). To facilitate comparison with previously estimated divergence times, we used an *Afzelia* fossil dated to 27.23 Ma (Pan et al., 2010) as in Donkpegan et al. (2017). Since our RAxML phylogeny was unable to resolve the positioning of *Intsia* with respect to *Afzelia* and because of the similarity of species from both genera for morphological characters used to describe the fossil (Kadiri and Olowokudejo, 2008; Pan et al., 2010), we considered the fossil to represent the minimum age for diversification of the *Afzelia-Intsia* clade. Bayesian analyses were done using the following priors: an uncorrelated lognormal relaxed clock model, a Yule process of speciation which is adequate to analyze data at the interspecific level (Yule, 1925; Heled and Drummond, 2015), and the selected nucleotide substitution model. The MCMC analyses were run for 50,000,000 generations, sampling trees every 1,000 generations. To evaluate convergence and ensure sufficient effective sample sizes (ESS values) for all BEAST parameters, we used Tracer 1.6 (Rambaut and Drummond, 2016). Runs were combined with LogCombiner after removing the first 10,000 samples as burn-in. Maximum Clade Credibility trees were produced in TreeAnnotator 1.8 (Drummond and Rambaut, 2007) and plotted in FigTree 1.4.4^4^.

#### Species Tree Inference: Species Delimitation and Estimation of Divergence Times

To provide additional support for species delimitation in *Afzelia*, we used several models to estimate species trees directly from the *Afzelia dataset*. We used the multi-species coalescent approach in SNAPP v.1.3.0 (Bryant et al., 2012) implemented in BEAST2 v.2.5.2 (Bouckaert et al., 2014) to estimate species trees from a multilocus SNP matrix. SNAPP is based on a Bayes Factor Delimitation method (BFD^∗^) (Leaché et al., 2015) which allows for the comparison of alternative species delimitation models in an explicit multispecies coalescent framework. The corresponding VCF file was converted to PHYLIP format using the Python script “vcf2phylip”^5^ and the XML input file for SNAPP analyses was prepared using the Ruby script “snapp_prep.rb”^6^. Two independent Markov-Chain Monte Carlo (MCMC) simulations were run for one million generations each, sampling trees at 1000 step intervals. Stationarity and convergence of chains were visually checked in TRACER v1.6 (ESS > 1000; Rambaut and Drummond, 2016)^7^. The program DensiTree v.2.2.6 (Bouckaert and Heled, 2014) was used to visualize the SNAPP trees after discarding the first 10% of each MCMC chain as burn-in. The resulting tree and log files were combined with Logcombiner 1.8.2^8^ with a burn-in of 10 000 for each run. A maximum-clade-credibility summary tree was generated with TreeAnnotator (Drummond and Rambaut, 2007) and visualized in FigTree v.1.4.3^9^. Divergence dating based on the multispecies coalescent has been shown to provide more accurate results than dating of concatenation-based species trees for the ages of younger nodes, which are commonly overestimated when concatenation is used (Stange et al., 2018). We dated the resulting species tree in SNAPP using the same fossil as above. To generate a SNAPP input XML file for divergence dating, we followed the protocol of Stange et al. (2018), using their provided Ruby script (“snapp_prep.rb”). According to their approach, a molecular clock and effective population sizes are shared between all species. We assumed the age of the root to be within a normal distribution of mean = 27.23 Ma and standard deviation being 10% of that variation, with an offset of 20 Ma.

We then used the Generalized Mixed Yule Coalescent (GMYC) model (Pons et al., 2006; Fujisawa and Barraclough, 2013) based on a likelihood method for species delimitation in *Afzelia*. To account for uncertainty in species delimitation, we used the single (*sGMYC*) and multiple (*mGMYC*) threshold species delimitation models using packages APE (Paradis et al., 2004) and SPLITS (Ezard et al., 2013) in R (R Core Team, 2017). The GMYC method requires an ultrametric tree (i.e., calibrated with a molecular clock), which was constructed using BEAST v.1.8 (Bouckaert et al., 2014). We used a relaxed log-normal clock with a coalescent tree prior as these have been identified as best prior parameters for GMYC analyses (Esselstyn et al., 2012). Monte Carlo Markov chains (MCMC) were run for one million iterations, sampling every 1000 iterations. Convergence of chains was assessed using Tracer v.1.6 (Rambaut and Drummond, 2016). The consensus tree (maximum clade credibility tree; 10% burn in; tree not presented) was constructed with TreeAnnotator v.1.7 (Drummond and Rambaut, 2007).

Finally, we used Bayesian (*bPTP*) and ML (*mlPTP*) implementations of the Poisson tree processes model (PTP) (available at http://species.h-its.org/ptp/) to estimate the number of speciation events in the *Afzelia* rooted phylogenetic tree based on nucleotide substitutions (Zhang et al., 2013). Because this approach does not require ultrametrization of trees, it constitutes a reasonable alternative to other species delimitation models such as the General mixed Yule coalescent model (Pons et al., 2006). In PTP models, the numbers of substitutions (branch lengths) represent speciation or branching events and, therefore, they only require a phylogenetic tree as input. PTP analyses were conducted on the web server for PTP (available at http://species.h-its.org/ptp/) using the best ML tree resulting from the RA × ML analysis (see below).

### Inference of Interspecific Hybridization History

We used TreeMix v1.12 (Pickrell and Pritchard, 2012) to infer historical relationships among *Afzelia* species. This method builds a maximum likelihood graph that connects species with their common ancestor, using the covariance structure of allele frequencies between species and a Gaussian approximation for genetic drift. Migration events, i.e., hybridization events, can be modeled to improve the fit of the inferred graph. To meet TreeMix requirements, the *Afzelia dataset* was reduced to SNPs without missing data, a single SNP was selected per GBS locus using VCFtools, and species-level allele frequencies were computed from the VCF to generate the TreeMix infile. We modeled the interspecific evolutionary history in *Afzelia* using *m* = 0 to *m* = 4 migration events.

## Results

### GBS Data Production and Reference Sequence Construction

Unambiguous barcodes were found in a total of 295 million sequencing reads. After trimming, cleaning, and quality filtering an average of 4.7 million reads per accession were retained. Genotyping *Afzelia* accessions with PyRAD yielded accession-level heterozygosity estimates between 0.0288 and 0.0794 and error rates between 0.0043 and 0.0169 (Supplementary Material S3). The accession with the lowest amount of missing data in the PyRAD genotyping – AD657 of *A. bipindensis* – was used as a GBS reference sequence. It comprised a total of 221,334 loci representing 3,489,577 bp, including 52,314 polymorphic sites, and 3749 scaffolds (Supplementary Material S3). This reference is available in FASTA format in DRYAD^10^.

### Mapping and SNP Calling

Mapping the reads from all accessions against the reference and genotyping with GATK allowed us to obtain VCF files for two datasets. For the *Afzelia with outgroups* dataset 21,150 SNPs were discovered and 9,165 SNPs were retained in 26 *Afzelia* accessions and all seven outgroups, after filtering INDELs, non-biallelic sites, and sites with more than 40% of missing data. For the *Afzelia* dataset 23,694 SNPs were discovered and 4,823 SNPs were retained (26 accessions) after filtering INDELs, non-biallelic sites, and sites with more than 20% of missing data. The *Afzelia with outgroups* and *Afzelia* datasets were used to generate phylogenetic trees in RAxML. For species delimitation based on the multispecies coalescent in SNAPP, a subset of 2,370 bi-allelic SNPs without missing data in at least one species was retained.

### Concatenation-Based Tree and Timing of *Afzelia* Diversification

**FIGURE 2:**
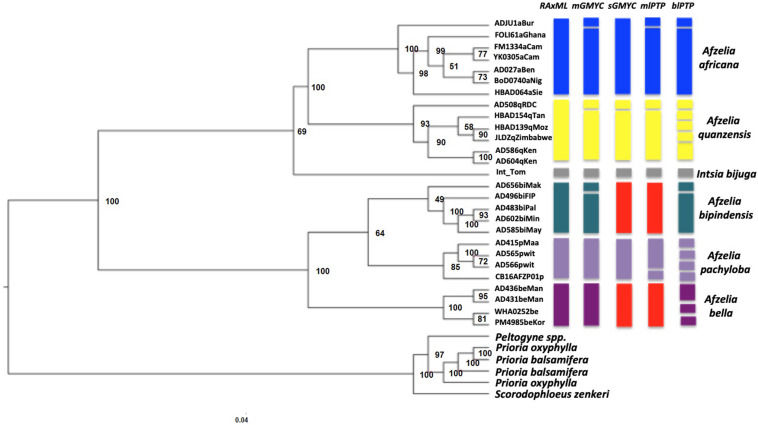
Phylogenetic relationships in *Afzelia* and related taxa inferred from nuclear genomic data. Maximum likelihood tree (33 samples, 9165 SNPs) estimated in RaxML. The tree was created using a 50% majority rule consensus tree from 500 bootstrap replicates. The consensus multilocus coalescent species trees of the genus *Afzelia* based on four models are represented in the right part of the figure using colored blocks. Each botanically determined species is indicated with the same branch color as in Figure 1. Each separate block stands for a separate lineage or taxonomic entity as delimited with the species delimitation model noted above. The red color representing *A. bipindensis* and *A. bella* in sGMYC and mlPTP indicates that the two species shared the same clade for these models.

For the *Afzelia with outgroups* dataset, different datasets (on the percentage of missing data) were tested for phylogenomics of the genus. The dataset (9,165 SNPs) of at most 40% missing data yielded the phylogenetic relationships that were most congruent with the known topology in the legumes (Bruneau et al., 2008; LPWG, 2017). The *Afzelia* clade (including *Intsia bijuga*) formed a monophyletic group in the RAxML tree (Figure 2). We also obtained two strongly supported monophyletic clades that correspond to the diploid and tetraploid lineages in the *Afzelia-Intsia* clade. All *Afzelia* species – represented by multiple accessions each – appeared monophyletic with strong support for four species and lower support for *A. bipindensis*. Such well-supported species delimitation had never been found in previous studies of phylogeny in the genus, especially in the rainforest clade of tetraploid species. The differentiation of the savannah species was well supported. In contrast, the topology of the diversification of the three rainforest species was not well resolved, as revealed by low bootstrap supports. The analyses with ascertainment bias correction to avoid overestimation of branch lengths and biases in the phylogeny using SNP datasets, resulted in identical topology and did not improve the resolution of the rainforest clades (results not shown).

**FIGURE 3:**
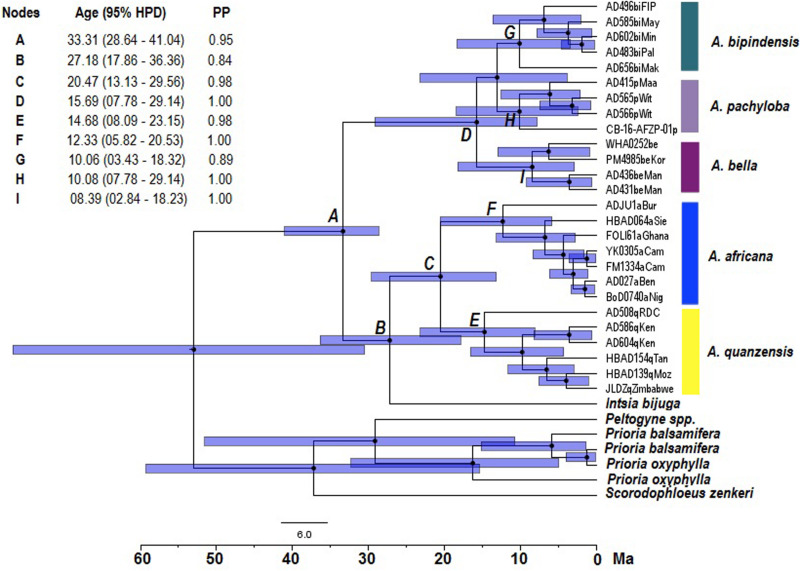
Divergence time chronograms obtained from the Bayesian maximum clade credibility tree reconstructed with 26 accessions of *Afzelia*, one *Intsia bijuga* and six outgroup accessions based on 9165 SNPs. The age of nodes associated with letters, the 95% highest posterior density (HPD) and the posterior probability (PP) are given in the insert. Bars indicate the 95% HPD intervals around node ages.

Based on molecular dating of the concatenation-based phylogeny, diversification appeared to occur earliest in the diploid lineage of *Afzelia*, while the position of the sister species *Intsia bijuga* remained ambiguous on the topology of the tree (Figure 3). The diversification of the *Afzelia-Intsia* clade started in the Oligocene, the posterior mean age of the common ancestor (MRCA, node A) of the clade being estimated at 33.31 Ma [95% highest posterior density (HPD) 28.64–41.04 Ma] (Figure 3). The divergence of each monophyletic species on the basis of the well-resolved phylogenetic tree at the genus level suggests that savannah species diversified in the Middle Miocene, *A. africana* (node F, 12.33 Ma) and *A. quanzensis* (node E, 14.68 Ma), whereas the rainforest species would have appeared in the Upper Miocene: *A. bipindensis* (node G, 10.06 Ma), *A. pachyloba* (node H, 10.08 Ma) and *A. bella* (node I, 08.39 Ma).

### Coalescent-Based Species Trees and Timing of *Afzelia* Diversification

**FIGURE 4:**
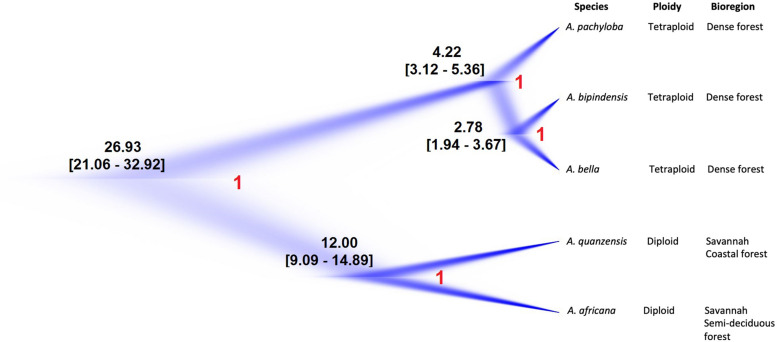
Nuclear species tree estimation in *Afzelia* based on 2370 SNPs as inferred by SNAPP and summary of species’ ecological and ploidy characteristics. Maximum-clade-credibility tree is shown in dark blue. Estimated ages have been indicated at each node with 95% highest posterior density (HPD) and posterior probability (in red).

For the *Afzelia* dataset the SNAPP analysis resolved five well-differentiated clades that support the monophyly of all species. The visualization of the species trees superimposed in the DensiTree plot (Figure 4) resolved the evolutionary relationships among species with no signs of conflict among trees, even within the rainforest clade: *A. pachyloba* is the sister species of the clade containing *A. bipindensis* and *A. bella*. The results of the *GMYC* and *PTP* models are plotted against the RAxML phylogeny in Figure 2. They resolved between 6 and 15 sub-lineages within *Afzelia*. The *sGMYC* and *mlPTP* models placed *A. bella* and *A. bipindensis* into the same species cluster, in line with the close relationship revealed by the SNAPP species tree. The SNAPP-calibrated tree revealed, as expected, later diversification dates than the concatenated gene tree. The diversification of *Afzelia* has a the posterior mean age of the MRCA in the late Oligocene at 26.93 Ma (95% HPD 21.06 – 32.92 Ma; Figure 4). The rainforest lineage diversified rapidly between the Pliocene and the early Pleistocene (mean *A. pachyloba* split at 4.22 Ma and mean *A. bipindensis*/*A. bella* split at 2.78 Ma) and the savannah lineage earlier in the Miocene (mean at 12 Ma).

### Interspecific Hybridization History

**FIGURE 5:**
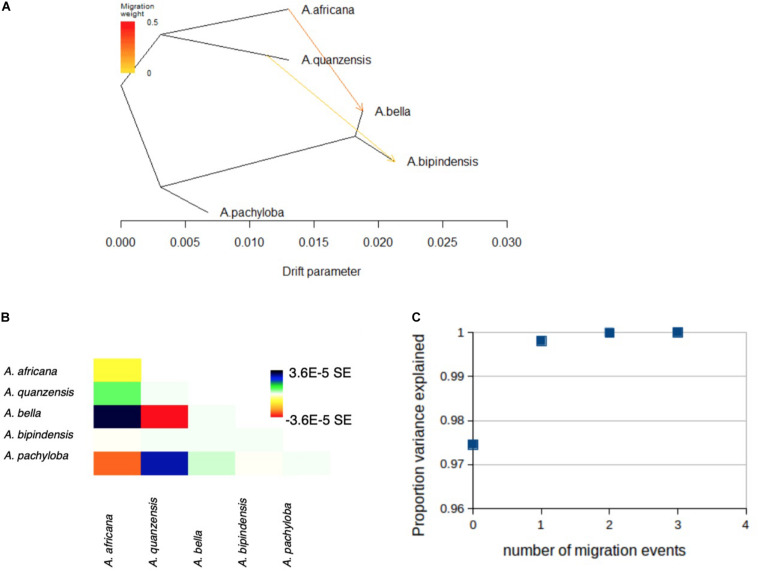
Evolutionary history among *Afzelia* species as inferred by TreeMix. **(A)** Graph showing the topology and branch lengths according to drift parameter, allowing for *m* = 2 migration events, represented by arrows. **(B)** Residual fit for the graph shown in **(A)**. The residual covariance between each pair of species scaled by the average standard error across all pairs is plotted. Colors are described in the palette on the right. Residuals above zero (green and blue) represent species that are more closely related to each other in the data than in the best-fit tree, and thus are candidates for admixture events. **(C)** Proportion of variance of the data explained by the four models run in TreeMix using *m* = 0 to *m* = 4 migration events.

The genetic relationships among species revealed by TreeMix distinguished the diploid and tetraploid clades, in agreement with the phylogenetic relationships evidenced with concatenation or coalescent-based methods, and confirmed the placement of *A. pachyloba* as sister of the clade containing *A. bipindensis* and *A. bella*, as revealed by species delimitation methods (Figure 5A). The proportion of variance in the data explained by the model was high, PVE = 0.975, for a model without migration. The addition of migration events improved the proportion of variance explained to PVE = 0.998 for *m* = 1 and PVE = 0.9999 for *m* = 2 migration events. The first migration event links diploid *A. africana* with tetraploid *A. bella* whereas the second links an ancestor of diploid *A. quanzensis* with tetraploid *A. bipindensis* (Figure 5).

## Discussion

Our phylogenetic reconstructions provided the most robust phylogenetic framework of the tropical tree genus *Afzelia* in Africa produced to date. Both the SNP concatenated gene tree (RAxML tree; Figure 2) and the coalescent-based species tree (SNAPP tree; Figure 4) highly supported two major monophyletic clades associated with habitat and ploidy levels: a diploid savannah clade and a tetraploid rainforest clade. The calibrated phylogeny and the species tree (Figures 3, 4) show an earlier diversification of the savannah clade followed by a later speciation within the rainforest clade. Species delimitation within these two major clades, with all species resolved as monophyletic, was also the most robust to date.

### A Single Biome Shift in African *Afzelia*

Genotyping-by-sequencing data strongly supported the monophyly of two major habitat-specific clades in *Afzelia*. This suggests a single transition between the savannah and the rainforest biomes in Africa. Previous plastid DNA sequence data – Sanger sequences of a few loci and full plastome sequences – placed the savannah species *A. quanzensis* as sister to the rainforest clade (Donkpegan et al., 2017). In contrast, the phylogenetic analyses of the multilocus genomic GBS data strongly supported the monophyly of the savannah clade and suggested a unique forest-savannah shift in the diversification of *Afzelia* in Africa.

The calibrated phylogeny and the dated species tree indicate a diversification of the savannah clade in the Miocene, earlier than the rainforest clade. This early diversification of the savannah species is exceptional in the context of the global plant evolutionary patterns reported in tropical Africa and South America that point to a relatively young age for savannah lineages. In the South African tree flora, the majority of divergence times between sister taxa of savannah trees were dated within the Pleistocene (the last 2 Ma), which is more recent than those between sister taxa of forest trees (Maurin et al., 2014). In the Brazilian savannah (Cerrado) the origin of woody plants restricted to the Cerrado is also estimated to be recent (<10 Ma), most of them in the Pliocene (<4 Ma; Simon et al., 2009). This seems to reflect the relative ages of the biomes, with rainforests dating from at least the early Paleocene (Burnham and Johnson, 2004; Jacobs et al., 2010), whereas savannahs are much younger, arising only in the Miocene (Jacobs, 2004; Senut et al., 2009; Simon et al., 2009; Pennington and Hughes, 2014).

Evolutionary shifts of plant lineages from the rainforest to savannah or to other dry biomes seem to have been significantly more frequent than switches of plant lineages into the rainforest (Simon et al., 2009; Simon and Pennington, 2012, Donoghue and Edwards, 2014, Maurin et al., 2014, Tosso et al., 2018; Freitas et al., 2019; Monthe et al., 2019). It seems logical that older biomes, i.e., the rainforests, will act as sources of lineages for younger biomes, i.e., the savannahs. Of course, there is no *a priori* reason to expect biome switches to be unidirectional, and given enough time, savannah lineages may re-enter rainforests. This might have been the case of the African *Afzelia*, where molecular phylogenies indicate recent splits within the rainforest lineage in the Upper Miocene and Pleistocene. If we assume that the splits of extant species occurring in the same biome indicate a shared ancestor within that biome, it is possible that the African *Afzelia* originated as a diploid savannah lineage followed by whole genome duplication and subsequent diversification in the rainforest. However, we should note that biome conservatism is more common than biome shifts in phylogenies of Southern Hemisphere plants (Crisp et al., 2009) and that the topology of the *Afzelia* phylogeny alone did not allow us to conclude on the direction of the biome shift, as both monophyletic clades diversified into their respective rainforest or savannah biomes.

In terms of morphological trait variation in *Afzelia*, dry forest species (*A. africana* and *A. quanzensis*) are clearly differentiated from rainforests species (*A. bella*, *A. bipindensis*, and *A. parviflora*) based on vegetative and floral variables (Donkpegan et al., 2017). The main morphological discriminant variables which separate dry forest versus rainforest species are (i) the rounded versus truncated-attenuated basal leaflet shape; (ii) at the distal end of the leaflets, the scalloped versus acuminate and mucronate shapes (sometimes with black spots). Leaves are longer and have a smaller number of secondary veins in dry forest vs. rainforest *Afzelia* species (Donkpegan et al., 2017). It is, however, difficult to associate these leaf and vein trait differences with a clear habitat-adaptive role in the light of the literature (Sack and Scoffoni, 2013; Tng et al., 2013) and to our knowledge there are no studies available that treat functional or physiological trait variation in *Afzelia*.

### Evolutionary Radiation of the African Rainforest Species

Previous Sanger sequencing of two nuclear and three plastid regions revealed extensive allele sharing across the rainforest clade of *Afzelia*. Accessions of the same species were scattered across the trees (Donkpegan et al., 2017), which could be due to incorrect taxonomy, recent speciation or ongoing hybridization. The GBS data effectively resolved species delimitation and diversification times of *Afzelia* in the rainforest. All species – which are morphologically similar and broadly sympatric- were resolved as monophyletic, validating the latest taxonomic revisions. The data also revealed a recent diversification process, and no trace of hybridization between species.

In *Afzelia*, autopolyploidization occurred prior to rapid speciation in the rainforests, suggesting the role of whole-genome duplications in the onset of adaptive radiations. Polyploidy represents an immediate source of genetic novelty that may promote evolutionary changes and divergence (Wood et al., 2007). Rapid speciation events immediately after polyploidization are well known in the evolution of plant groups and point at the success of whole-genome duplications as triggers of speciation (Soltis et al., 2007). The rainforest *Afzelia* species correspond to monophyletic clades, although the clade support varied depending on the filtering parameters used to generate the datasets as well as on the phylogenetic method chosen. This weaker phylogenetic support suggests incomplete lineage sorting in the tetraploid species, which is consistent with their more recent diversification and the larger effective population sizes of tetraploid organisms (Arnold et al., 2012). In addition, the stronger support for monophyly in the savannah than rainforest species is in agreement with similar observations in South America (Pennington and Lavin, 2016). The pattern of strong support for monophyletic species in seasonally dry tropical forests in South America has been attributed to maintenance of resident lineages adapted to a stable, seasonally dry ecology; conversely lower support for monophyly in rainforest trees was attributed to lesser habitat stability creating opportunities for immigration and speciation from taxa with large effective population sizes extending over large areas (Pennington and Lavin, 2016; Heuertz et al., 2020).

### Coalescent-Based Phylogenetics and Population Genetics of Multiple Nuclear Loci for Species Delimitation in Recent Radiations

In the rainforest clade of *Afzelia*, phylogenomic analysis revealed short branches among species, in line with a scenario of recent radiation, and revealed the superior performance of the coalescent-based over the concatenation-based methods. Using concatenation-based trees, the topology changed depending on the filtering parameters, which might be explained by the poor performance of this approach under highly incomplete lineage sorting, typically found in cases of rapid speciation (Fernández-Mazuecos et al., 2018). Coalescent-based species trees are based on tests of alternative hypotheses of species delimitation. In this context, lineages do not need to be resolved as monophyletic in gene trees, which leads to more reliable estimates of the evolutionary relationships and divergence times. Overall, an approach combining multiple nuclear loci with coalescent-based phylogenetic analyses was revealed as the optimal approach to resolve and date the radiation of the rainforest clade.

Forcing the rainforest radiation into bifurcating phylogenetic trees may be problematic because of the age of the radiation and potential for interspecific gene flow. Therefore, we explored the possibility of reticulate evolution using a population genetics approach implemented in TreeMix. Using genome-wide SNPs we found no evidence for gene flow between rainforest species despite their rapid genetic differentiation and sympatric distribution. Given the limited sample sizes within species it is possible that we have missed introgression events limited to sympatric populations of both species. Nevertheless, our analyses identified possible hybridization events that led to incorporation of genetic variation from savannah to rainforest species after the split of the main lineages. Such hybridization events could potentially explain the previously observed cyto-nuclear incongruence, where *A. quanzensis* grouped with rainforest species for plastid DNA but with *A. africana* for two nuclear regions (Donkpegan et al., 2017). *Afzelia quanzensis* could thus have captured plastid DNA from rainforest species during an ancient hybridization event, or else, incomplete lineage sorting may explain the pattern. Altogether, phylogenetic evidence in *Afzelia* points to the existence of hybridization and introgression between species despite differences in ploidy, as has been observed in other plant lineages, such as orchids of the genus *Epidendrum* (Pinheiro et al., 2010) or grasses of the genus *Spartina* (Ainouche et al., 2009).

Similar to Donkpegan et al. (2017) our study did not permit to solve the phylogenetic position of *Intsia bijuga*, the putative sister clade to *Afzelia*. *Intsia bijuga* was placed within the *Afzelia* clade, confirming the close relatedness of both genera. Multilocus data of Asian *Afzelia* species and increased taxon sampling in *Intsia* with multiple accessions per species may help improve the phylogenetic positioning of *Intsia*.

## Conclusion

We have elucidated the evolutionary history of the widespread emblematic and threatened African tree species of the genus *Afzelia* using genome-wide multilocus data. While the genus was previously recognized as a species complex (Donkpegan et al., 2015), we showed that based on our set of accessions and markers, all species were resolved as monophyletic, and that diversification in the savannah clade preceded that of the rainforest clade. Phylogenomic studies thus represent promising approaches to clarify evolutionary relationships in taxonomic groups that show a high level of variation.

## Data Availability Statement

Fasta reference sequence is available from the Dryad data repository: https://datadryad.org/stash/share/QK6Ay8vq7grepB66aGWp42Ir8PCwjrpSR2IXUqX4rRk. Raw fastq sequences of GBS data are being submitted to GenBank’s Sequence Read Archive.

## Author Contributions

AD, MH, and RP conceived the study. All authors collected the data, performed the analyses, interpreted the results, and contributed to drafting and writing the manuscript.

## Conflict of Interest

The authors declare that the research was conducted in the absence of any commercial or financial relationships that could be construed as a potential conflict of interest.

## Footnotes

1 https://github.com/najoshi/sabre

2 http://sourceforge.net/projects/picard/files/picard-tools/1.96/

3 www.phylo.org

4 http://tree.bio.ed.ac.uk/software/figtree/

5 https://github.com/edgardomortiz/vcf2phylip

6 https://github.com/mmatschiner/snapp_prep

7 http://beast.bio.ed.ac.uk/tracer/

8 http://beast.bio.ed.ac.uk/LogCombiner

9 http://tree.bio.ed.ac.uk/software/figtree/

10 https://doi.org/10.5061/dryad.95~x~69p8gf

## Supplementary Material

MATERIAL S1 Sample origins (fresh and herbarium material) of plant tissue samples used for the phylogenetic analyses of *Afzelia. “*na” means that no collector or voucher names are mentioned on the specimen consulted and studied.

MATERIAL S2 The parameters used in PyRAD for *de novo* assembly.

MATERIAL S3 Results from *de novo* assembling of 30 *Afzelia* and *Intsia* accessions. (nloci) is the number of loci identified in the intra-accession clustering; (f1loci) is the number of loci with >N depth coverage; (f2loci) number of loci with >N depth and passed paralog filter; (nsites) number of sites across f loci; (npoly) number of polymorphic sites in nsites; (poly) frequency of polymorphic sites. H and E are heterozygosity and error rate, respectively.
