# Supplementary material for: Miocene Diversification in the Savannahs Precedes Tetraploid Rainforest Radiation in the African Tree Genus Afzelia (Detarioideae, Fabaceae)
Source: Front Plant Sci. 2020 Jun 17;11:798. doi: 10.3389/fpls.2020.00798 (PMC7313659; doi:10.3389/fpls.2020.00798)
Supplement: MATERIAL S1 — Sample origins (fresh and herbarium material) of plant tissue samples used for the phylogenetic analyses of Afzelia. “na” means that no collector or voucher names are mentioned on the specimen consulted and studied. [file Table_1.docx]

**SUPPLEMENTARY MATERIAL**

**Supplementary Material S1.**

| **Species** | **Country** | **Locality** | **Vouchers** | **Collectors** | | **Herba-rium** | **Longitude** | **Latitude** |
| --- | --- | --- | --- | --- | --- | --- | --- | --- |
| *A. bipindensis* | Cameroon | na | 786biMva | Donkpegan S.L.A. | | BRLU | 11.58262 | 2.6379 |
| *A. africana* | Benin | Lama | AD027aBen | Donkpegan S.L.A. | | BRLU | 2.14591 | 6.97603 |
| *A. africana* | Senegal | Casamense | AD123aSen | Donkpegan S.L.A. | | BRLU | 12.493559 | -16.2198 |
| *A. africana* | Togo | Notse | AD248aTog | Donkpegan S.L.A. | | BRLU | 1.289516 | 6.959194 |
| *A. bipindensis* | Cameroon | Mindourou | AD298biPal | Donkpegan S.L.A. | | BRLU | 14.362304 | 3.271999 |
| *A. bipindensis* | Cameroon | Maan | AD380biMaa | Donkpegan S.L.A. | | BRLU | 10.470714 | 2.259002 |
| *A. bella* | Cameroon | Maan | AD436beMan | Donkpegan S.L.A. | | BRLU | 9.566711 | 5.743841 |
| *A. africana* | Burkina-Faso | Comoe | AD488aBur | Tosso F. | | BRLU | 4.62765 | 9.8248 |
| *A. bipindensis* | Cameroun | Fipcam | AD489biFIP | Monthe F. | | BRLU | 13.60438 | 3.98363 |
| *A. quanzensis* | DRC | Lubembe | AD508qRDC | Procces P. | | BRLU | 22.532668 | -10.9204 |
| *A. quanzensis* | Kenya | Gede | AD518qKen | Donkpegan S.L.A. | | BRLU | 39.98244 | -3.27098 |
| *A. pachyloba* | Gabon | Mayumba | AD565pwit | Donkpegan S.L.A. | | BRLU | 10.781703 | -3.25323 |
| *A. bipindensis* | Gabon | Mayumba | AD585biMay | Donkpegan S.L.A. | | BRLU | 10.835241 | -3.28078 |
| *A. bipindensis* | Cameroon | Mindourou | AD602biMin | Donkpegan S.L.A. | | BRLU | 13.35395 | 3.58518 |
| *A. bipindensis* | Gabon | Bambidie | AD630biBam | Donkpegan S.L.A. | | BRLU | 12.900413 | -0.718511 |
| *A. bipindensis* | Gabon | Makokou | AD656biMak | Donkpegan S.L.A. | | BRLU | 13.68172 | 0.905028 |
| *A. africana* | Nigeria | Akure | BoD0744aNig | Demenou B. | | BRLU | 5.083333 | 7.25 |
| *A. pachyloba* | Congo | Nyanga | CB16AFZP01p | Bourland N. | | BRLU | 11.9118 | -2.5027 |
| *A. africana* | Cameroon | Ngambetica | FM1301aCam | Monthe F. | | BRLU | 11.29621 | 4.78896 |
| *A. africana* | Ghana | Ejura | FOLI61aGhana | na | | BRLU | 1.18467 | 7.2993 |
| *A. africana* | Siera-Leone | na | HBAD064aSie | Cole E.A. | | WAG | 8.216667 | -12.333 |
| *A. quanzensis* | Mozambique | na | HBAD139qMoz | Koning J. de | | WAG | -25.05 | 33.39 |
| *A. quanzensis* | Tanzania | na | HBAD154qTan | na | | BRLU | -4.031399 | 35.29907 |
| *Intsia bijuga* | Madagascar | Tamatave | HBAD32/1469 | Schatz G.E & D’arcy W. | | BR | 49.13333 | -18.95 |
| *A. bipindensis* | Congo | Loundoungou | JFG0741biLou | Gillet J-F. | | BRLU | 17.0628 | 2.384 |
| *A. bipindensis* | Congo | Pokola | JFG0865biPok | Gillet J-F. | | BRLU | 16.4 | 1.33 |
| *A. quanzensis* | Zimbabwe | Victoria Falls | JLDZqZimbabwe | Doucet J-L. | | BRLU | -17.9333 | 25.8333 |
| *A. bella* | Cameroon | Korup | PM4985beKor | Parmentier I. | | BRLU | 8.854 | 5.062 |
| *A. bella* | Ghana |  | WHA0252be | na | | BRLU | -2.00293 | 4.92232 |
| *A. bipindensis* | Gabon | Makokou | AD657biMak | Donkpegan S.L.A. | | BRLU | 13.673089 | 0.906143 |
| *A. pachyloba* | Cameroon | Maan | AD415pMaa | Donkpegan S.L.A. | | BRLU | 10.472715 | 2.228791 |
| *A. bella* | Cameroon | Maan | AD431beMan | Donkpegan S.L.A. | | BRLU | 9.560244 | 5.730079 |
| *A. bipindensis* | Cameroon | Mindourou | AD483biPal | Donkpegan S.L.A. | | BRLU | 14.07295 | 3.02891 |
| *A. bipindensis* | Cameroon | Fipcam | AD496biFIP | Monthe F. | | BRLU | 13.62304 | 3.99228 |
| *A. pachyloba* | Gabon | Mayumba | AD566pwit | Donkpegan S.L.A. | | BRLU | 10.78101 | -3.25393 |
| *A. quanzensis* | Kenya | Witu | AD586qKen | Donkpegan S.L.A. | | BRLU | 40.47216 | -2.37142 |
| *A. quanzensis* | Kenya | Witu | AD604qKen | Donkpegan S.L.A. | | BRLU | 40.48026 | -2.37063 |
| *A. bipindensis* | Gabon | Bambidie | AD620biBam | Donkpegan S.L.A. | | BRLU | 12.96966 | -0.75242 |
| *A. africana* | Burkina-Faso | Pama | ADJU1aBur | Linchant J. | | BRLU | 0.78157 | 11.2512 |
| *A. africana* | Nigeria | Bolorunduro | BoD0740aNig | Demenou B. | | BRLU | 5.5036 | 7.8627 |
| *A. africana* | Cameroon | Sibati | YK0305aCam | Kamga Y. | | BRLU | 12.79475 | 6.30011 |
| *A. africana* | Cameroon | Ngambetica | FM1334aCam | Monthe F. | | BRLU | 11.33028 | 5.15375 |
| *Scorodophloeus zenkeri* | Gabon | Makokou | AD0700_c5a | Donkpegan S.L.A. | | BRLU | 13.67273 | 0.90746 |
| *Prioria oxyphylla* | DRC | Baume | DK0085oxyCDR | Kaviri D. | | BRLU | 25.42013 | 1.00302 |
| *Prioria balsamifera* | Gabon | Bambidie | BAM16balGAB | Donkpegan S.L.A. | | BRLU | 12.96812 | -0.75340 |
| *Prioria oxyphylla* | Congo | Mokabi Dzanga | JFG0196oxyCOG | Gillet J-F. | | BRLU | 16.74757 | 3.2765 |
| *Peltogyne sp 1* | Guyana | Saut_Lavilette SE of Regina | LV109332ATF        Chave J. & Grosso B. | | | Chave’s lab | na | na |
| *Prioria balsamifera* | Gabon | Makokou | MAK1balGAB | | Donkpegan S.L.A. | BRLU | 13.67780 | 0.901026 |

**Supplementary Material S2.**


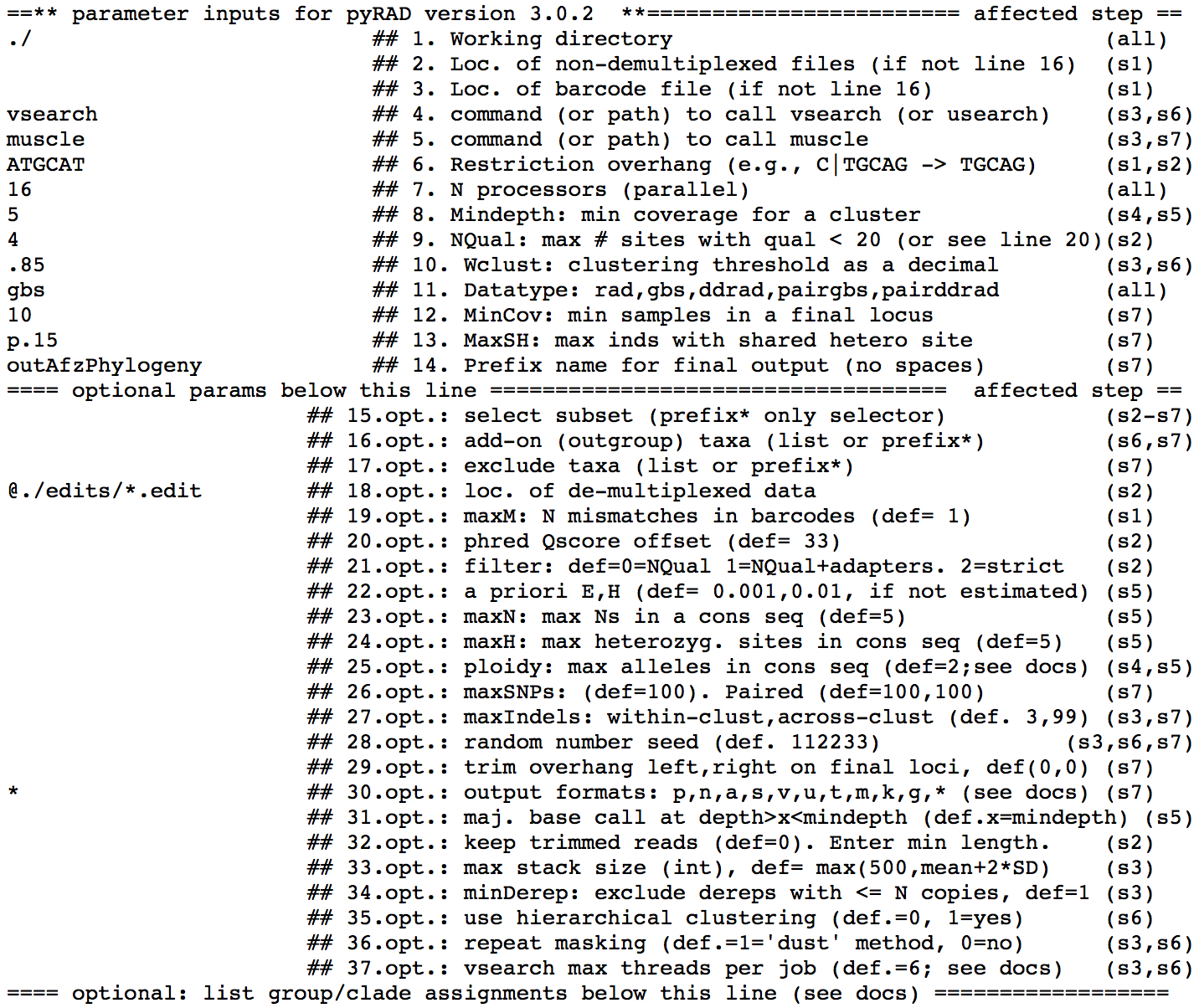


**Supplementary Material S3.**

| **Species** | **Vouchers** | **nloci** | **f1loci** | **f2loci** | **nsites** | **npoly** | **poly** | **H** | **E** |
| --- | --- | --- | --- | --- | --- | --- | --- | --- | --- |
| *A. bipindensis* | 786biMva | 61445 | 16897 | 11999 | 931798 | 5879 | 0.0063 | 0.0313 | 0.0094 |
| *A. africana* | AD027aBen | 148721 | 54435 | 38579 | 3330121 | 14429 | 0.0043 | 0.0282 | 0.0042 |
| *A. africana* | AD123aSen | 81211 | 27434 | 19455 | 1638491 | 6742 | 0.0041 | 0.0319 | 0.0056 |
| *A. africana* | AD248aTog | 61107 | 14972 | 10219 | 849197 | 3541 | 0.0042 | 0.0358 | 0.0097 |
| *A. bipindensis* | AD298biPal | 114997 | 34284 | 22261 | 1776471 | 20097 | 0.0113 | 0.0367 | 0.0083 |
| *A. bipindensis* | AD380biMaa | 133856 | 44483 | 28765 | 2370340 | 17970 | 0.0076 | 0.0300 | 0.0063 |
| *A. bella* | AD436beMan | 139580 | 55880 | 32408 | 2696279 | 35436 | 0.0131 | 0.0335 | 0.0057 |
| *A. africana* | AD488aBur | 83694 | 28966 | 20557 | 1779746 | 7107 | 0.0040 | 0.0309 | 0.0060 |
| *A. bipindensis* | AD489biFIP | 128475 | 45327 | 29893 | 2481563 | 17984 | 0.0072 | 0.0288 | 0.0060 |
| *A. quanzensis* | AD508qRDC | 38935 | 9005 | 8183 | 704153 | 2194 | 0.0031 | 0.0144 | 0.0008 |
| *A. quanzensis* | AD518qKen | 112686 | 39516 | 27435 | 2410808 | 12575 | 0.0052 | 0.0308 | 0.0066 |
| *A. pachyloba* | AD565pwit | 33970 | 5603 | 3735 | 285067 | 1612 | 0.0057 | 0.0398 | 0.0104 |
| *A. bipindensis* | AD585biMay | 118935 | 38754 | 24695 | 2025170 | 15392 | 0.0076 | 0.0299 | 0.0065 |
| *A. bipindensis* | AD602biMin | 137547 | 45040 | 25273 | 2051993 | 18087 | 0.0088 | 0.0293 | 0.0063 |
| *A. bipindensis* | AD630biBam | 124030 | 37822 | 24535 | 1974072 | 21864 | 0.0111 | 0.0351 | 0.0080 |
| *A. bipindensis* | AD656biMak | 130825 | 44631 | 27215 | 2177400 | 27200 | 0.0125 | 0.0342 | 0.0066 |
| *A. africana* | BoD0744aNig | 138401 | 52429 | 37692 | 3221605 | 13189 | 0.0041 | 0.0291 | 0.0043 |
| *A. pachyloba* | CB16AFZP01p | 19280 | 1074 | 445 | 33157 | 496 | 0.0150 | 0.0565 | 0.0228 |
| *A. africana* | FM1301aCam | 1216 | 40 | 17 | 1389 | 12 | 0.0086 | 0.0794 | 0.0169 |
| *A. africana* | FOLI61aGhana | 109238 | 43935 | 31090 | 2713187 | 11624 | 0.0043 | 0.0293 | 0.0050 |
| *A. africana* | HBAD064aSie | 46836 | 10995 | 7958 | 637513 | 2710 | 0.0043 | 0.0342 | 0.0094 |
| *A. quanzensis* | HBAD139qMoz | 60134 | 18418 | 13363 | 1117030 | 5471 | 0.0049 | 0.0323 | 0.0078 |
| *A. quanzensis* | HBAD154qTan | 71218 | 22829 | 16211 | 1307802 | 7459 | 0.0057 | 0.0329 | 0.0082 |
| *Intsia bijuga* | HBAD32/1469 | 100629 | 26084 | 15881 | 1241670 | 15777 | 0.0127 | 0.0428 | 0.0122 |
| *A. bipindensis* | JFG0741biLou | 64832 | 12133 | 8845 | 706385 | 5322 | 0.0075 | 0.0339 | 0.0083 |
| *A. bipindensis* | JFG0865biPok | 37772 | 7345 | 5050 | 392221 | 2380 | 0.0061 | 0.0375 | 0.0097 |
| *A. quanzensis* | JLDZqZimbabwe | 160536 | 67725 | 47428 | 4184049 | 27978 | 0.0067 | 0.0313 | 0.0039 |
| *A. bella* | PM4985beKor | 121355 | 41247 | 25208 | 1977658 | 25236 | 0.0128 | 0.0365 | 0.0076 |
| *A. bella* | WHA0252be | 161143 | 61512 | 36894 | 3133355 | 21847 | 0.0070 | 0.0250 | 0.0035 |
| *A. bipindensis* | AD657biMak | 221334 | 76359 | 41722 | 3489577 | 52314 | 0.0150 | 0.0310 | 0.0063 |
